# Maternal dietary patterns during pregnancy and birth weight: a prospective cohort study

**DOI:** 10.1186/s12937-024-01001-8

**Published:** 2024-08-28

**Authors:** Tongtong Li, Yusa He, Nan Wang, Chengwu Feng, Puchen Zhou, Ye Qi, Zhengyuan Wang, Xiaojun Lin, Dou Mao, Zhuo Sun, Aili Sheng, Yang Su, Liping Shen, Fengchang Li, Xueying Cui, Changzheng Yuan, Liang Wang, Jiajie Zang, Geng Zong

**Affiliations:** 1 CAS Key Laboratory of Nutrition, Metabolism and Food Safety, Shanghai Institute of Nutrition and Health, University of Chinese Academy of Sciences, Chinese Academy of Sciences, 320 Yue-yang Rd, Shanghai, 200031 China; 2 Department of Epidemiology & Biostatistics, School of Public Health, Zhejiang University, Hangzhou, 310058 Zhejiang China; 3 Division of Health Risk Factors Monitoring and Control, Shanghai Municipal Center for Disease Control and Prevention, Shanghai, 200336 China; 4 School of Public Health, the Second Affiliated Hospital, Zhejiang University School of Medicine, Hangzhou, 310058 Zhejiang China; 5 Department of Big Data in Health Science School of Public Health, Center of Clinical Big Data and Analytics of The Second Affiliated Hospital, Zhejiang University School of Medicine, Hangzhou, Zhejiang China; 6 Department of Nutrition, Shanghai Chest Hospital, Shanghai Jiao Tong University, Shanghai, 200030 China; 7 Department of Public Health, Marshall University, West Virginia, USA; 8 Marshall Global Health Institute, Marshall University, West Virginia, USA

**Keywords:** Plant-based dietary pattern, Macrosomia, Birth weight, Chinese

## Abstract

**Background:**

Existing data on maternal dietary patterns and birth weight remains limited and inconsistent, especially in non-Western populations. We aimed to examine the relationship between maternal dietary patterns and birth weight among a cohort of Chinese.

**Methods:**

In this study, 4,184 mother-child pairs were included from the Iodine Status in Pregnancy and Offspring Health Cohort. Maternal diet during pregnancy was evaluated using a self-administered food frequency questionnaire with 69 food items. Principal component analysis was used to identify dietary patterns. Information on birth weight and gestational age was obtained through medical records. Adverse outcomes of birth weight were defined according to standard clinical cutoffs, including low birth weight, macrosomia, small for gestational age, and large for gestational age.

**Results:**

Three maternal dietary patterns were identified: plant-based, animal-based, and processed food and beverage dietary patterns, which explained 23.7% variance in the diet. In the multivariate-adjusted model, women with higher adherence to the plant-based dietary patten had a significantly higher risk of macrosomia (middle tertile vs. low tertile: odds ratio (OR) 1.45, 95% CI 1.00-2.10; high tertile vs. low tertile: OR 1.55, 95% CI 1.03–2.34; *P*-trend = 0.039). For individual food groups, potato intake showed positive association with macrosomia (high tertile vs. low tertile: OR 1.72, 95% CI 1.20–2.47; *P*-trend = 0.002). Excluding potatoes from the plant-based dietary pattern attenuated its association with macrosomia risk. No significant associations was observed for the animal-based or processed food and beverage dietary pattern with birth weight outcomes.

**Conclusions:**

Adherence to a plant-based diet high in carbohydrate intake was associated with higher macrosomia risk among Chinese women. Future studies are required to replicate these findings and explore the potential mechanisms involved.

**Supplementary Information:**

The online version contains supplementary material available at 10.1186/s12937-024-01001-8.

## Introduction

As the fundamental indicator of fetal development, birth weight has been associated with various health outcomes throughout the life span, including perinatal mortality, cognitive and behavioral development, and chronic diseases in adulthood [1–5]. Abnormal birth weight has been a global public health issue over the last decades. For example, low birth weight (LBW) accounts for 15–20% of live births overall, of which 91.0% are in low-to-middle income countries [6, 7], while the prevalence of macrosomia has also been rapidly (10–15%) growing [8, 9]. In this regard, studies on potential risk factors of abnormal birth weights are needed [10].

Maternal diet during pregnancy plays a crucial role in determining birth weight [11, 12], and high intakes of fruits, vegetables, whole grains, low-fat dairy products, vegetable oils, and fish have been strongly associated with birth weight [13]. However, existing evidence was mostly based on Western countries, and little is known about the situation in the rest of the world [11, 13, 14]. Plant-based diets, which are common among Asians, have been associated with larger birth sizes in this population (+ 40.5 g, *P* = 0.01), compared to inverse associations among Europeans [15–18]. One study in South Americans reported that a diet pattern with high intake of fast food and sweets was associated with over a 4-fold higher odds of large for gestational age (LGA) [19], yet adherence to a processed food and beverage dietary pattern during pregnancy was associated with a 9.4-fold higher risk of LBW in Asians [20]. Collectively, these equivocal findings call for further investigations regarding the role of maternal diet on birth weight.

With a rapid socioeconomic shift, many Asian countries experienced drastically changing landscapes for diet and health-related issues including a rapid decline in birth rates in several areas observed recently [21–23]. In China, a trend from the traditional plant-based diet pattern to a high-fat diet has characterized dietary change that is also shared by other Asian countries [24–26], while existing data showed the prevalence of LBW and macrosomia remain high (5.2% and 6.9% respectively) [8, 27]. Studies on maternal diet and birth outcomes in this population are thus needed, yet findings remain scarce and inconsistent [13]. In this regard, we derived major maternal dietary patterns among a large sample of pregnant women in Shanghai, a well-developed metropolitan in China, and examined their associations with key birth weight outcomes, such as small for gestational age (SGA), LGA, LBW, and macrosomia.

## Methods

### Study population and design

In 2017, 5,042 pregnant women were enrolled in the Iodine Status in Pregnancy and Offspring Health Cohort (ISPOHC) study, which used a multistage, stratified random sampling method to obtain a representative sample of pregnant women from all 16 districts in Shanghai [28]. Specifically, each district was subdivided into five sampling areas, with one street randomly chosen from each area, and 40 to 70 pregnant women selected from each street. Participants were involved during the whole pregnancy period, with 1/3 recruited in each trimester. At baseline, they completed initial questionnaires that collected detailed information on demographics, medical history, lifestyle, and medications, as well as a 69-item food frequency questionnaire (FFQ). In this study, participants were excluded if they were without a live, singleton infant (*n* = 76), with implausibly high or low energy intake (less than 500 kcal/day or more than 5,000 kcal/day; *n* = 295), with gestational diabetes or hypertension during the pregnancy at baseline (*n* = 307), or with missing values on offspring birth weight (*n* = 180), leaving 4,184 women for analysis.

ISPOHC obtained ethical approval from the Ethics Committee of the Shanghai Centre for Disease Control and Prevention. All participants provided informed consent through a written signature at baseline assessment.

### Dietary assessment and dietary patterns

Food intake during the previous three months of baseline was assessed by trained staff through face-to-face interviews [29]. The semi-quantitative FFQ was adapted from a longer version that has been used for nutrition surveys in Shanghai, and the validation results have been reported elsewhere [30]. We converted food frequencies into daily intakes (g/day) and energy intake (kcal/day) using the China Food Composition Database [31], and further aggregated the 69 food items into 25 non-overlapping food groups, based on nutrients or common characteristics (Table S1) [32]. When combining food items, such as soy milk and tofu, we converted wet weight into dry weight based on conversion factors provided in the China Food Composition Database (Table S2) [31].

After the Bartlett test of sphericity and Kaiser-Mayer-Olkin tests, we used principal component analysis (PCA) with varimax rotation to characterize maternal dietary patterns. The number of components (dietary patterns) retained was based on a Scree plot, eigenvalue, and meaningful interpretation of the patterns. Dietary patterns were named based on food groups with high factor loadings (*r* > 0.4). We calculated dietary pattern scores for each participant by summing the standardized intakes of food groups (g/day) weighted by their factor loadings. Higher dietary pattern scores indicated greater adherence to the pattern. The dietary pattern scores were further divided into tertile 1 (reference), tertile 2, and tertile 3 for regression analyses.

### Outcome ascertainment

The primary outcomes of the study were LBW and macrosomia, and the secondary outcomes were SGA and LGA. Birth weight in grams and gestational age in weeks was obtained through medical records. The newborns were classified as LBW (birth weight < 2,500 g), normal birth weight (birth weight 2,500-4,000 g), and macrosomia (birth weight > 4,000 g) based on standard clinical cutoffs [33]. Moreover, SGA was defined as birth weight below the 10th percentile of sex-and-gestational-age-specific birth weight, while LGA was defined as above 90th percentile, according to the China neonatal growth standards for gestational age of 24–42 weeks [34]. Infants with gestational age beyond this range were excluded from the SGA and LGA analysis (*n* = 2 excluded).

### Covariate assessment

Baseline questionnaires collected information on maternal age, baseline season, parity, gestational periods, pre-pregnancy weight, maternal education, household income, alcohol drinking, smoking, physical activity, and uses of micronutrient supplementation for multivitamin, calcium, and folic acid. Baseline height (cm) was measured by trained staffs, and pre-pregnancy body mass index (BMI) was calculated as pre-pregnancy weight (kg)/height squared (m^2^) [35]. Maternal domicile place was identified using national ID number, and grouped as south China, north China, and Shanghai surrounding area. Physical activities were measured using the long format, the Chinese version of the International Physical Activity Questionnaire, and sufficient leisure-time physical activity was defined as at least 150 min/week of moderate activity or 75 min/week of vigorous activity or an equivalent combination [36]. Passive smoking was defined as being exposed to second-hand smoking for at least one day per week. Infant sex and gestational age at birth in weeks were obtained at delivery.

### Statistical analysis

Maternal characteristics were summarized according to the tertiles of each diet pattern. Continuous variables were presented as mean (SD), and categorical variables were described as proportions. Missing values were imputed using medians or modes if missing rate of the covariates was < 5%, otherwise, coded as an independent category. Detailed information on missing rates and imputation methods of covariates can be found in Table S3.

We built three multivariate logistic regression models to estimate the odds ratios (ORs) and 95% confidence intervals (95% CIs) of the association between dietary pattern scores and birth weight outcomes. Model 1 was adjusted for maternal age (continuous variables, in years) and infant sex (male or female). Model 2 further included maternal domicile place (south China, north China, or Shanghai surrounding area), pre-pregnancy BMI (< 18.5, 18.5–23.9, or ≥ 24.0 kg/m^2^), household income (< 100,000, 100,000-350,000, or ≥ 350,000 yuan/year), education (< 13 or ≥ 13 years), baseline season (spring/winter, summer, or autumn), parity (primiparous or multiparous), gestational periods at recruitment (first, second, or third trimester), passive smoking (yes or no), alcohol drinking (yes or no), physical activity (active or inactive), uses of multivitamin supplements (yes or no), uses of calcium supplements (yes or no), uses of folic acid supplements (yes or no), and total energy intake (continuous variables, in kcals/day). Model 3 was mutually adjusted for other dietary patterns based on model 2. For LBW and macrosomia, we further adjusted for gestational age at birth (continuous variables, in weeks) in the final model. *P* for trend was obtained by modeling the median values of diet pattern score in each tertile into the models.

When significant associations between dietary patterns and birth outcomes were observed, we further examined the associations between food groups and birth outcomes using logistic regression models after adjusting for the same set of covariates used in model 2 of the main analysis.

We conducted prespecified subgroup analyses by maternal age (< 29 or ≥ 29 years, median), gestational periods at baseline (first, second, or third trimester), passive smoking (yes or no), physical activity (active or inactive), and infant sex (male or female). In this analysis, diet patterns and birth weight were modeled as continuous variables. Potential effect modification was tested in a model that includes diet patterns, stratification factors (categorical variables), and their interaction terms, with *P* values of the interaction term used as tests for significance.

Several sensitivity analyses were conducted. First, we limited analyses to women with spontaneous labor to exclude the influence of delivery mode. Second, we performed analyses in women with term (born between 37 to < 42 completed weeks) infants. Third, we excluded women of were less than six weeks pregnant at assessment to improve the accuracy of dietary intake during pregnancy. Fourth, we assessed whether imputation methods affected the findings by repeating analyses after multiple imputations of missing data in covariates using chained equations. We created 5 imputed datasets and pooled the estimates from logistic regression models across imputed datasets [37]. Lastly, for food groups that showed significant associations with birth weight outcomes, we conducted a sensitivity analysis by leaving it out of the PCA to test the robustness of the findings.

A two-sided *P* value < 0.05 was considered statistically significant. All statistical analyses were performed in R (v4.2.1, R Foundation for Statistical Computing).

## Results

### Diet patterns

PCA identified three major dietary patterns, and the variance in diet explained was 8.90% for the plant-based dietary pattern, 8.48% for the animal-based dietary pattern, and 6.31% for the processed food and beverage dietary pattern (Table 1). The ranges of dietary pattern scores (Table S4), average intakes of individual food groups according to diet pattern tertiles (Table S5), intercorrelations of food intakes (Figure S1), and differences in food intake per one unit of diet pattern score (Table S6) were presented in the supplementary material. The plant-based pattern was characterized by high consumption of fruits and vegetables, potatoes, and refined and whole grains. Women in the animal-based pattern consumed relatively high amounts of fish, red meat, and poultry. Processed food and beverage pattern was loaded for processed meats and vegetables, sauce, tea and coffee.

**Table 1:** Structures of three dietary patterns extracted by principal component factor analysis among 4,184 pregnant women in Shanghai, China

Food Groups	Plant-based dietary pattern	Animal-based dietary pattern	Processed food and beverage dietary pattern
Other fruits	**0.61**	0.05	-0.04
Potato	**0.51**	0.08	0.12
Berries	**0.49**	0.07	-0.06
Refined grains	**0.48**	0.01	0.22
Other vegetables	**0.45**	0.33	0.08
Whole grain	**0.43**	0.04	0.04
Oranges	**0.43**	-0.10	-0.04
Legumes and legume products	0.33	0.23	0.14
Nuts	0.32	0.18	-0.04
Leafy vegetables	0.32	0.32	-0.03
Freshwater seafood	0.06	**0.66**	-0.12
Seafood	-0.01	**0.65**	0.04
Red Meat	0.11	**0.59**	0.20
Poultry	0.09	**0.55**	0.21
Fungi and algae	0.22	0.31	0.10
Dairy	0.12	0.28	-0.16
Eggs	0.16	0.24	-0.14
Rice	-0.04	0.15	0.02
Processed meat	0.12	0.10	**0.55**
Sauce	0.04	-0.02	**0.46**
Tea	-0.11	0.12	**0.45**
Coffee	-0.14	0.11	**0.43**
Pickled vegetables	0.07	-0.05	**0.42**
Fried food	0.09	-0.02	0.38
Pastry	0.24	-0.02	0.29
Proportion of explained variance (%)	8.90	8.48	6.31

Factor loading represents the relative contribution of each food group to the dietary pattern. The food groups whose factor loading > 0.4 in each dietary pattern are shown in bold characters

### Baseline characteristics

Baseline characteristics of the participants by dietary patterns were presented in Table 2. Women with higher plant-based diet pattern were more likely to come from north China, with passive smoking, sufficient physical activity, and higher intakes of multivitamins and calcium supplements, and higher energy from carbohydrates. They were more likely to be recruited in summer with later trimesters. Women with higher scores of the animal-based dietary pattern were more likely to be born in Shanghai and surrounding areas, with higher childbearing age, incomes and education, passive smoking, lower physical activities, using multivitamins and folic acids, and more energy from fat and protein. They were more likely to be recruited in summer and autumn during the third trimester. Women with higher scores of the processed food and beverage diet pattern were more likely to be with higher pre-pregnancy BMI, less passive smoking, more alcohol drinking, physical activity, uses of multivitamins, and with more energy from carbohydrate and fat. They were more likely to be recruited during winter, being nulliparous, and with male infants.

**Table 2:** Baseline characteristics of participants by dietary pattern scores among pregnant women in Shanghai, China

		Plant-based dietary pattern				Animal-based dietary pattern				Processed food and beverage dietary pattern			
	Overall	Tertile 1	Tertile 2	Tertile 3	*P*	Tertile 1	Tertile 2	Tertile 3	*P*	Tertile 1	Tertile 2	Tertile 3	*P*
N	4184	1395	1394	1395		1395	1394	1395		1395	1394	1395	
Maternal age, mean (SD), years	29.5 (4.4)	29.5 (4.4)	29.6 (4.5)	29.4 (4.2)	0.428	29.1 (4.4)	29.7 (4.4)	29.8 (4.3)	< 0.001	29.5 (4.4)	29.6 (4.3)	29.4 (4.4)	0.630
Maternal domicile place, n (%)					< 0.001				0.012				0.045
North China	699 (16.7)	185 (13.3)	220 (15.8)	294 (21.1)		263 (18.9)	233 (16.7)	203 (14.6)		239 (17.1)	228 (16.4)	232 (16.6)	
Shanghai surrounding area	2727 (65.2)	942 (67.5)	927 (66.5)	858 (61.5)		864 (61.9)	919 (65.9)	944 (67.7)		931 (66.7)	879 (63.1)	917 (65.7)	
South China	758 (18.1)	268 (19.2)	247 (17.7)	243 (17.4)		268 (19.2)	242 (17.4)	248 (17.8)		225 (16.1)	287 (20.6)	246 (17.6)	
Baseline season, n (%)					< 0.001				< 0.001				0.033
Spring/winter	814 (19.5)	287 (20.6)	256 (18.4)	271 (19.4)		360 (25.8)	280 (20.1)	174 (12.5)		262 (18.8)	299 (21.4)	253 (18.1)	
Summer	1334 (31.9)	394 (28.2)	436 (31.3)	504 (36.1)		418 (30.0)	426 (30.6)	490 (35.1)		421 (30.2)	437 (31.3)	476 (34.1)	
Autumn	2036 (48.7)	714 (51.2)	702 (50.4)	620 (44.4)		617 (44.2)	688 (49.4)	731 (52.4)		712 (51.0)	658 (47.2)	666 (47.7)	
Maternal parity, multiparous, n (%)	1735 (41.5)	581 (41.6)	558 (40.0)	596 (42.7)	0.347	573 (41.1)	592 (42.5)	570 (40.9)	0.646	531 (38.1)	564 (40.5)	640 (45.9)	< 0.001
Gestational periods, n (%)					0.019				< 0.001				0.106
First trimester	1387 (33.2)	505 (36.2)	456 (32.7)	426 (30.5)		498 (35.7)	494 (35.4)	395 (28.3)		430 (30.8)	463 (33.2)	494 (35.4)	
Second trimester	1627 (38.9)	517 (37.1)	533 (38.2)	577 (41.4)		576 (41.3)	509 (36.5)	542 (38.9)		549 (39.4)	547 (39.2)	531 (38.1)	
Third trimester	1170 (28.0)	373 (26.7)	405 (29.1)	392 (28.1)		321 (23.0)	391 (28.0)	458 (32.8)		416 (29.8)	384 (27.5)	370 (26.5)	
Pre-pregnancy BMI, kg/m^2^, n (%)					0.352				0.215				0.014
< 18.5	558 (13.3)	205 (14.7)	179 (12.8)	174 (12.5)		209 (15.0)	171 (12.3)	178 (12.8)		185 (13.3)	208 (14.9)	165 (11.8)	
18.5–23.9	2961 (70.8)	967 (69.3)	986 (70.7)	1008 (72.3)		978 (70.1)	994 (71.3)	989 (70.9)		1000 (71.7)	985 (70.7)	976 (70.0)	
≥ 24.0	665 (15.9)	223 (16.0)	229 (16.4)	213 (15.3)		208 (14.9)	229 (16.4)	228 (16.3)		210 (15.1)	201 (14.4)	254 (18.2)	
Household income, yuan/year, n (%)					0.752				< 0.001				0.108
< 100 000	697 (16.7)	237 (17.0)	218 (15.6)	242 (17.3)		295 (21.1)	204 (14.6)	198 (14.2)		225 (16.1)	231 (16.6)	241 (17.3)	
100 000–350 000	2983 (71.3)	992 (71.1)	1001 (71.8)	990 (71.0)		967 (69.3)	1007 (72.2)	1009 (72.3)		981 (70.3)	1019 (73.1)	983 (70.5)	
≥ 350 000	504 (12.0)	166 (11.9)	175 (12.6)	163 (11.7)		133 (9.5)	183 (13.1)	188 (13.5)		189 (13.5)	144 (10.3)	171 (12.3)	
Maternal education, < 13 years, n (%)	1277 (30.5)	427 (30.6)	405 (29.1)	445 (31.9)	0.263	495 (35.5)	406 (29.1)	376 (27.0)	< 0.001	396 (28.4)	434 (31.1)	447 (32.0)	0.092
Passive smoking, no, n (%)	2463 (58.9)	858 (61.5)	827 (59.3)	778 (55.8)	0.008	801 (57.4)	854 (61.3)	808 (57.9)	0.081	907 (65.0)	823 (59.0)	733 (52.5)	< 0.001
Alcohol drinking status, no, n (%)	3773 (90.2)	1270 (91.0)	1260 (90.4)	1243 (89.1)	0.217	1251 (89.7)	1259 (90.3)	1263 (90.5)	0.730	1289 (92.4)	1255 (90.0)	1229 (88.1)	0.001
Leisure-time physical activity, active, n (%)	1698 (40.6)	492 (35.3)	587 (42.1)	619 (44.4)	< 0.001	513 (36.8)	581 (41.7)	604 (43.3)	0.001	535 (38.4)	565 (40.5)	598 (42.9)	0.052
Multivitamin, yes, n (%)	1742 (41.6)	506 (36.3)	613 (44.0)	623 (44.7)	< 0.001	517 (37.1)	578 (41.5)	647 (46.4)	< 0.001	538 (38.6)	595 (42.7)	609 (43.7)	0.015
Calcium, yes, n (%)	1678 (40.1)	499 (35.8)	571 (41.0)	608 (43.6)	< 0.001	561 (40.2)	554 (39.7)	563 (40.4)	0.941	559 (40.1)	555 (39.8)	564 (40.4)	0.946
Folic acid, yes, n (%)	2589 (61.9)	846 (60.6)	846 (60.7)	897 (64.3)	0.074	913 (65.4)	845 (60.6)	831 (59.6)	0.003	891 (63.9)	846 (60.7)	852 (61.1)	0.168
Total energy intake, mean (SD), kcal/day	1881.5 (720.9)	1478.0 (541.0)	1791.5 (545.1)	2375.0 (744.9)	< 0.001	1574.4 (621.0)	1766.6 (584.4)	2303.6 (739.3)	< 0.001	1834.6 (664.0)	1724.5 (640.4)	2085.4 (800.1)	< 0.001
Carbohydrate, E%, mean (SD)	53.7 (8.6)	49.7 (9.2)	53.6 (7.3)	57.9 (7.0)	< 0.001	53.6 (9.6)	53.8 (8.1)	53.7 (8.0)	0.871	53.2 (8.6)	53.1 (8.6)	54.9 (8.4)	< 0.001
Protein, E%, mean (SD)	16.0 (4.7)	15.2 (5.4)	16.0 (4.2)	16.7 (4.3)	< 0.001	12.4 (2.6)	15.3 (2.6)	20.2 (4.7)	< 0.001	16.1 (4.8)	15.0 (4.3)	16.7 (4.9)	< 0.001
Fat, E%, mean (SD)	30.2 (9.4)	35.1 (10.1)	30.4 (7.8)	25.3 (7.3)	< 0.001	33.9 (10.4)	30.8 (8.1)	26.0 (7.7)	< 0.001	30.6 (9.1)	31.9 (9.7)	28.3 (8.9)	< 0.001
Infants sex, male, n (%)	2145 (51.3)	725 (52.0)	725 (52.0)	695 (49.8)	0.417	696 (49.9)	722 (51.8)	727 (52.1)	0.447	753 (54.0)	709 (50.9)	683 (49.0)	0.028
Gestational age at birth, mean (SD), weeks	38.9 (1.4)	38.9 (1.3)	38.9 (1.4)	38.9 (1.4)	0.750	39.0 (1.4)	38.9 (1.3)	38.9 (1.4)	0.231	38.9 (1.4)	39.0 (1.3)	38.9 (1.4)	0.312

Data are mean (SD) or n (%), unless otherwise specified

### Dietary patterns in relation to birth weight traits

In total, 105 (2.5%) infants were born with LBW and 219 (5.2%) with macrosomia. In the maternal age and infant sex adjusted model (Table 3), a higher plant-based dietary pattern score was significantly associated with higher risks of macrosomia, and the findings remained after multivariate adjustment in model 2 and 3. The adjusted odds ratio (OR) for the highest tertile compared with the lowest was 1.55 (95% CI 1.03–2.34; *P*-trend = 0.039). Compared with individuals in the lowest tertile of animal-based dietary pattern score, those in the middle tertile had a higher risk of macrosomia (adjusted OR 1.72; 95% CI 1.20–2.45) but no significant trend was observed (*P*-trend = 0.502). The association between processed dietary pattern and LBW or macrosomia was not significant in any models.

**Table 3:** Associations between three dietary patterns and low birth weight and macrosomia among pregnant women in Shanghai, China

	Score category			
Variables	Tertile 1	Tertile 2	Tertile 3	*P*-trend
**Low birth weight**				
Plant-based dietary pattern				
N Cases/Total	36/1341	32/1315	37/1309	
Model 1	1.00 (ref)	0.90 (0.55–1.46)	1.06 (0.67–1.69)	0.745
Model 2	1.00 (ref)	0.78 (0.44–1.38)	0.74 (0.38–1.43)	0.383
Model 3	1.00 (ref)	0.80 (0.45–1.44)	0.76 (0.38–1.52)	0.493
Animal-based dietary pattern				
N Cases/Total	28/1335	38/1303	39/1327	
Model 1	1.00 (ref)	1.38 (0.84–2.26)	1.38 (0.84–2.26)	0.251
Model 2	1.00 (ref)	1.71 (0.94–3.12)	1.70 (0.87–3.31)	0.167
Model 3	1.00 (ref)	1.57 (0.85–2.89)	1.44 (0.72–2.90)	0.403
Processed food and beverage dietary pattern				
N Cases/Total	44/1318	32/1333	29/1314	
Model 1	1.00 (ref)	0.71 (0.45–1.13)	0.66 (0.41–1.06)	0.109
Model 2	1.00 (ref)	0.79 (0.46–1.37)	0.55 (0.31-1.00)	0.054
Model 3	1.00 (ref)	0.84 (0.48–1.47)	0.59 (0.32–1.08)	0.073
**Macrosomia**				
Plant-based dietary pattern				
N Cases/Total	54/1359	79/1362	86/1358	
Model 1	1.00 (ref)	1.49 (1.05–2.13)	1.66 (1.17–2.36)	0.007
Model 2	1.00 (ref)	1.44 (0.99–2.07)	1.49 (0.99–2.24)	0.079
Model 3	1.00 (ref)	1.45 (1.00-2.10)	1.55 (1.03–2.34)	0.039
Animal-based dietary pattern				
N Cases/Total	60/1367	91/1356	68/1356	
Model 1	1.00 (ref)	1.56 (1.12–2.19)	1.14 (0.80–1.63)	0.766
Model 2	1.00 (ref)	1.69 (1.19–2.39)	1.17 (0.78–1.75)	0.723
Model 3	1.00 (ref)	1.72 (1.20–2.45)	1.22 (0.80–1.84)	0.502
Processed food and beverage dietary pattern				
N Cases/Total	77/1351	61/1362	81/1366	
Model 1	1.00 (ref)	0.79 (0.56–1.12)	1.07 (0.78–1.48)	0.435
Model 2	1.00 (ref)	0.77 (0.54–1.10)	0.98 (0.70–1.37)	0.862
Model 3	1.00 (ref)	0.82 (0.58–1.18)	1.07 (0.76–1.51)	0.673

ORs and 95% CIs were calculated in logistic model: model 1 adjusted for maternal age (continuous variables, in years), and infant sex (male or female); model 2 also included maternal domicile place (south China, north China, or Shanghai surrounding area), pre-pregnancy BMI (< 18.5, 18.5–23.9, or ≥ 24.0 kg/m^2^), household income (< 100,000, 100,000-350,000, or ≥ 350,000 yuan/year), education (< 13 or ≥ 13 years), baseline season (spring/winter, summer, or autumn), parity (primiparous or multiparous), gestational periods at recruitment (first, second, or third trimester), passive smoking (yes or no), alcohol drinking (yes or no), physical activity (active or inactive), multivitamin (yes or no), calcium tablets (yes or no), folic acid (yes or no), total energy (continuous variables, in kcal/day), and gestational week at birth (continuous variables, in weeks) on model 2. Model 3 mutually adjusted for all extracted dietary scores in the same model based on model 2

*P* for trend was obtained by modelling the median value of the tertiles into the logistic regression models

N Cases/Total = number of cases and total participants in the study. OR = odds ratio. CI = confidence interval. Ref = reference

In this study, 247 (5.9%) infants were born with SGA and 701 (16.8%) with LGA. No associations were observed between the three dietary patterns and SGA or LGA (Table S7).

### Individual food groups with macrosomia

Table S8 showed the associations between individual food groups highly related to the plant-based dietary pattern (*r* > 0.4) and macrosomia. In multivariable-adjusted logistic regression models, pregnant women in the highest tertile of potato intake were associated with a higher risk of having macrosomia infants, when compared to those of the lowest tertile (adjusted OR 1.72; 95% CI 1.20–2.47; *P*-trend = 0.002). No significant associations were observed between other components of the plant-based dietary pattern and macrosomia.

### Subgroup analyses

**Fig. 1:**
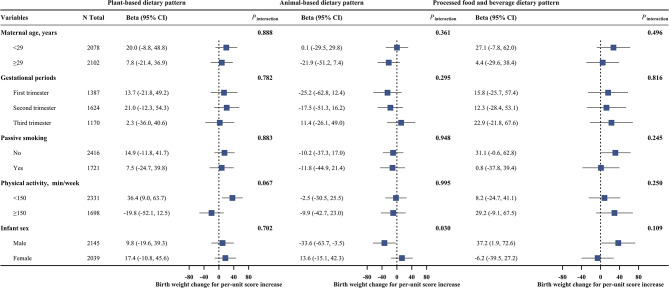
Subgroup analyses for the association between three dietary pattern scores (continuous, per 1-unit increase) and birth weight (continuous, in grams), stratified by maternal age, gestational periods, passive smoking, physical activity, and infant sex. Beta and 95% CI were calculated in linear model adjusted for maternal age (continuous variables, in years), and infant sex (male or female), maternal domicile place (south China, north China, or Shanghai surrounding area), pre-pregnancy BMI (< 18.5, 18.5–23.9, or ≥ 24.0 kg/m^2^), household income (< 100,000, 100,000-350,000, or ≥ 350,000 yuan/year), education (< 13 or ≥ 13 years), baseline season (spring/winter, summer, or autumn), parity (primiparous or multiparous), gestational periods at recruitment (first, second, or third trimester), passive smoking (yes or no), alcohol drinking (yes or no), multivitamin (yes or no), calcium tablets (yes or no), folic acid (yes or no), total energy (continuous variables, in kcal/day), gestational week at birth (continuous variables, in weeks), and the other two dietary scores. The corresponding stratification variable was excluded in the corresponding subgroup analysis, for example, infant sex was not adjusted in the analyses stratified by infant sex. N Total = number of total participants in the study. CI = confidence interval

In stratified analyses, higher birth weight associated with plant-based dietary pattern score turned to be observed in participants who did not reach the recommended level of physical activity (beta 36.4; 95% CI 9.0, 63.7 g per 1-unit increase in plant-based dietary pattern score) compared to those who reached the recommendation (beta − 19.8; 95% CI -52.1, 12.5 g per 1-unit increase in plant-based dietary pattern score; *P* for interaction = 0.067; Fig. 1). As for infant sex, a higher animal-based dietary pattern was associated with lower birth weight in males (beta − 33.6; 95% CI -63.7, -3.5 g per 1-unit increase in plant-based dietary pattern score; *P* for interaction = 0.030) rather than female. Consistent results were observed in analyses with stratification by maternal age, gestational periods at baseline, and passive smoking.

### Sensitivity analyses

**Fig. 2:**
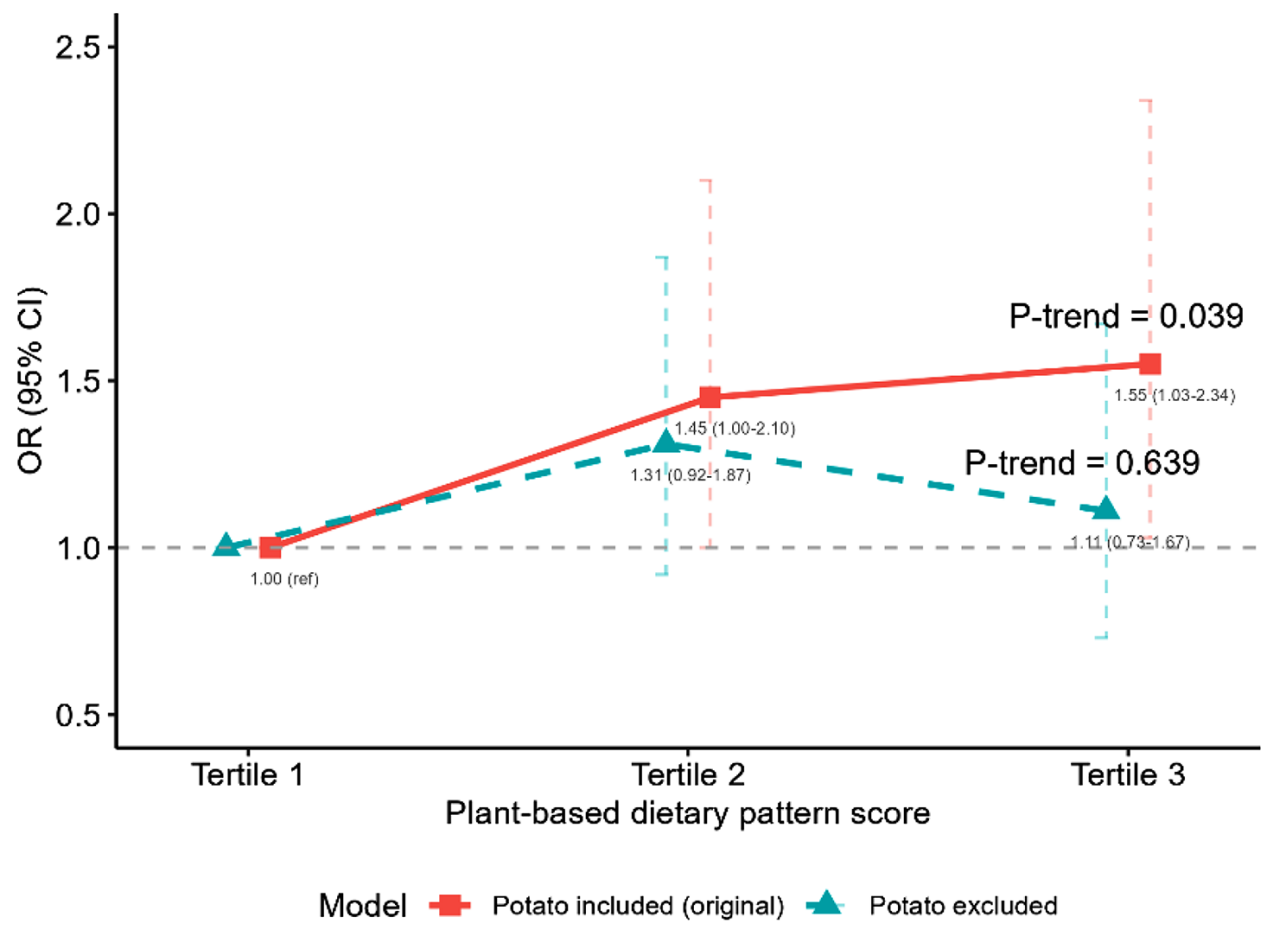
Associations between plant-based dietary pattern with and without potatoes and macrosomia. ORs and 95% CIs were calculated in logistic model adjusted for maternal age (continuous variables, in years), infant sex (male or female), maternal domicile place (south China, north China, or Shanghai surrounding area), pre-pregnancy BMI (< 18.5, 18.5–23.9, or ≥ 24.0 kg/m^2^), household income (< 100,000, 100,000-350,000, or ≥ 350,000 yuan/year), education (< 13 or ≥ 13 years), baseline season (spring/winter, summer, or autumn), parity (primiparous or multiparous), gestational periods at recruitment (first, second, or third trimester), passive smoking (yes or no), alcohol drinking (yes or no), physical activity (active or inactive), multivitamin (yes or no), calcium tablets (yes or no), folic acid (yes or no), total energy (continuous variables, in kcal/day), and gestational week at birth (continuous variables, in weeks) *P* for trend was obtained by modelling the median value of the tertiles into the logistic regression models OR = odds ratio. CI = confidence interval. Ref = reference

When we repeated the analysis among women with spontaneous labor (*N* = 2,231 with 83 cases of macrosomia), the association of plant-based dietary pattern score with macrosomia was attenuated (adjusted OR comparing top with bottom tertile 1.40; 95% CI 0.73–2.69; *P*-trend = 0.393; Table S9). The association remained when limiting the analysis to women with term infants (*N* = 4,001), to those with ≥ 6 weeks of gestation at baseline (*N* = 4,153), or when missing covariates were imputed (*N* = 4,184; Tables S10 to S12). After excluding potatoes, the association between the plant-based diet pattern and macrosomia was largely attenuated (Fig. 2).

## Discussion

In this study, three major dietary patterns, namely plant-based, animal-based, and processed food and beverage, were derived among pregnant women in China. We found that adherence to a dietary pattern rich in plant-based foods with high carbohydrate contents was associated with a higher risk of macrosomia. Potato intake was significantly associated with macrosomia and excluding it from the plant-based diet pattern attenuated the positive associations with macrosomia.

### Results in the context of other studies

Existing studies on the relationship between maternal dietary patterns and birth outcomes have mainly focused on low birth weight, rather than macrosomia, and shown large inconsistencies in findings [13, 38–41]. We reported a 55% higher risk for macrosomia associated with a higher adherence to a plant-based dietary pattern. However, in 3 studies of Europeans, the relative risks of macrosomia associated with high plant-based food intake were between 0.76 and 4.67 with high between-study heterogeneities (I^2^ = 95.9%) [13, 39]. One multiethnic study has shown that a plant-based diet in pregnancy was associated with larger birth sizes among Asians but not Europeans [42], yet in a study of 7,934 Chinese, a diet pattern similar to our plant-based dietary pattern was not associated with macrosomia (OR 1.17, 95% CI 0.93, 1.48]) [43]. Further studies are needed to explore potential reasons for inconsistencies in plant-based diet pattern and macrosomia before clinical recommendations are made.

The positive association between the plant-based diet and birth weight could be attributed to specific food groups loaded in this pattern. In our study, potato intake was associated with higher macrosomia risk, and excluding it from plant-based diet pattern attenuated the positive association with macrosomia. Potato consumption has been associated with certain risk factors of macrosomia, including gestational diabetes and long-term weight gain [44–46], which has been explained by hyperglycemia, insulin resistance, oxidative stress, and epigenetic modifications. For macrosomia, high carbohydrate foods that are heavily loaded in the plant-based diet pattern may increase glucose influx into the fetal circulation, elevating the fetal metabolic rate and excess fat deposition [44, 47, 48]. One previous study suggested a greater incidence of LGA births and higher birth weight in infants born to mothers consuming high-GI diets during pregnancy [49]. Furthermore, several studies found intakes of vegetables and fruits during pregnancy could be positively associated with birth weight [50, 51]. Collectively, these findings highlighted the potential role of high-carbohydrate foods in developing macrosomia, which needs to be confirmed by more evidence from experimental settings.

In our study, the positive association between the plant-based dietary pattern and birth weight turned to be mainly observed among individuals who did not meet the recommended physical activity level. Physical activity has been shown to prevent excessive gestational and fetal weight gain, possibly by balancing surplus energy from diet [52, 53]. One meta-analysis of 117 randomized clinical trials involving 34,546 pregnancies showed that participants not engaging in sufficient physical activity was associated with high risks of gestational diabetes, which is a known risk factor of fetal overgrowth [53–55]. In addition, we observed higher animal-based dietary pattern score was associated with lower birth weight among male infants rather than their female counterparts. Animal-based products (such as red meat and whole-fat dairy) are major contributors to a higher dietary inflammatory index [56], and a recent study found an inverse association between pro-inflammatory dietary score and birth weight only among male infants [33]. Clearly, more observational studies are needed to validate our findings and explore potential mechanisms for modifying role of physical activity or sex on diet and birth weight outcomes.

### Strengths and limitations

A strength of our study is that we used a large sample of pregnant women. Meanwhile, the study also has limitations. Firstly, based on the observational nature of the results, it is not possible to establish a cause-and-effect relationship. Secondly, although known risk factors and potential confounding factors were carefully considered, residual confounding including gestational weight gain cannot be ruled out. Thirdly, self-reported questionnaires were used to collect dietary and maternal information during pregnancy, which may have been susceptible to recall bias. Fourth, the FFQ used in our study is short and may not fully capture the complexity of diet, although the number of food items is comparable to other studies on maternal diet and birth outcomes among Chinese (61 to 64 items) [16, 57]. Fifth, PCA is based on the inter-correlations of major food groups, but is unable to distinguish healthy effects of components/food groups within a dietary pattern. For example, the potato was included in the plant-based pattern, while the processed food and beverage dietary pattern includes both processed foods with unhealthy potentials and tea/coffee that are often considered as healthy components of a diet. Finally, we could not capture potential changes in diet during pregnancy, although previous studies have suggested that maternal diets tend to be stable from preconception to the end of pregnancy [58, 59].

## Conclusions

In conclusion, we found that a plant-based diet pattern during pregnancy was associated with a higher risk of macrosomia, possibly owing to high carbohydrate content. Future work is required to replicate these findings in independent studies and to clarify the potential mechanisms that are responsible for these associations.

### Electronic supplementary material

Below is the link to the electronic supplementary material.

Supplementary Material 1

## Data Availability

No datasets were generated or analysed during the current study.

## Abbreviations

ISPOHC: Iodine Status in Pregnancy and Offspring Health Cohort
FFQ: Food frequency questionnaire
LBW: Low birth weight
SGA: Small for gestational age
LGA: Large for gestational age
GI: Glycemic index
PCA: Principal component analysis
