# Supplementary material for: Maternal dietary patterns during pregnancy and birth weight: a prospective cohort study
Source: Nutr J. 2024 Aug 28;23:100. doi: 10.1186/s12937-024-01001-8 (PMC11351029; doi:10.1186/s12937-024-01001-8)
Supplement: Supplementary file 1 — Supplementary Material 1 [file 12937_2024_1001_MOESM1_ESM.docx]

# Supplementary appendix

**Maternal dietary patterns during pregnancy and birth weight:**

**a prospective cohort study**

## Tables

### Table S1. Food groups used in the dietary pattern analysis.

| **Food groups** | **Items** |
| --- | --- |
| Rice | Rice |
| Refined grain | Noodles, bread, steamed buns, meat buns, vegetarian buns, dumplings, vermicelli |
| Potato | Potatoes |
| Fried food | Fried dough sticks and other fried foods |
| Pastry | Pastries (mooncakes, cakes, cookies, etc.) |
| Whole grain | Coarse grains and miscellaneous grains (oats, corn, barley) |
| Legume and legume products | Soybeans, mung beans, red beans, soy milk, tofu, dried tofu, other soy products (such as tofu skin and tofu bamboo) |
| Other vegetables | Legume Vegetables, fruiting vegetables, melon vegetables, onion, ginger and garlic, stem vegetables, root vegetables |
| Leafy vegetables | Cabbage vegetables, leafy vegetables |
| Fungi and algae | Black fungus, white fungus, shiitake mushrooms, seaweed, kelp, wakame, other edible fungi |
| Oranges | Oranges |
| Other fruits | Apples, pears, bananas, watermelons, other fruits (such as mangoes and pineapples) |
| Berries | Kiwi, grapes, strawberries |
| Dairy | Milk and yogurt |
| Nut | Walnuts, pine nuts and almonds, sunflower seeds, pumpkin seeds, peanuts |
| Red meat | Pork Belly, lean pork, pork chops, pork ribs, pork feet, beef and lamb |
| Poultry | Chicken and duck breast, whole chicken (duck), chicken (duck) wings, chicken (duck) legs |
| Freshwater seafood | Freshwater fish, river shrimp, freshwater crabs, freshwater clams |
| Seafood | Marine Fish, dried Shrimp, scallops, marine Shrimp, crab, other mollusks |
| Eggs | Eggs, duck eggs |
| Processed meat | Meat pickled products, brined meat products |
| Pickled vegetables | Pickled and salted vegetables |
| Sauce | Sauce |
| Tea | Tea |
| Coffee | Coffee |

### Table S2. Wet-to-dry weight conversion factors for food items

| **Food item (g)** | **Conversion factor** | **Converted to (g)** |
| --- | --- | --- |
| Tofu | 0.19 | Dry soybeans |
| Dried tofu | 0.45 | Dry soybeans |
| Tofu skin | 0.63 | Dry soybeans |
| Soy milk | 0.07 | Dry soybeans |
| Tofu bamboo | 1.43 | Dry soybeans |
| Kelp (wet) | 0.33 | Kelp (dry) |
| Wakame (wet) | 0.07 | Wakame (dry) |
| Black/white fungus (wet) | 0.14 | Black/white fungus (dry) |
| Shiitake Mushroom (wet) | 0.20 | Shiitake Mushroom (dry) |
| Tea (wet) | 0.02 | Tea (dry) |
| Coffee (wet) | 0.07 | Coffee (dry) |

Tofu skin and tofu bamboo were grouped into other soy products.

### Table S3. Missing rate and imputation method of covariates

| **Variable** | **Missing rate** | **Method** |
| --- | --- | --- |
| Age | 0.10% | Median |
| Infant sex | 0% | NA |
| Domicile place | 0.31% | Mode |
| Pre-pregnancy BMI | 1.22% | Mode |
| Household income | 0.29% | Mode |
| Education | 0.07% | Mode |
| Baseline season | 0.12% | Mode |
| Parity | 0.12% | Mode |
| Gestational periods at recruitment | 0.07% | Mode |
| Passive smoking | 1.12% | Mode |
| Alcohol drinking | 0.53% | Mode |
| Physical activity | 3.71% | Mode |
| Multivitamin | 6.69% | Missing category |
| Calcium tablets | 5.50% | Missing category |
| Folic acid | 2.15% | Mode |
| Total energy | 0% | NA |
| Gestational week at birth | 0% | NA |

NA=not applicable.

### Table S4. Distribution of three dietary pattern scores

|  | **Minimum** | **Maximum** | **Median (IQR)** |
| --- | --- | --- | --- |
| Plant-based dietary pattern score | -3.65 | 8.64 | -0.19 (1.12) |
| Animal-based dietary pattern score | -3.25 | 8.27 | -0.21 (1.01) |
| Processed food and beverage dietary pattern score | -3.21 | 10.03 | -0.25 (0.74) |

SD, standard deviation. IQR, interquartile range.

### Table S5. Food intakes according to tertiles of dietary pattern scores

|  |  | **Plant-based dietary pattern** | | | **Animal-based dietary pattern** | | | **Processed food and beverage dietary pattern** | | |
| --- | --- | --- | --- | --- | --- | --- | --- | --- | --- | --- |
|  | **Overall** | **Tertile 1** | **Tertile 2** | **Tertile 3** | **Tertile 1** | **Tertile 2** | **Tertile 3** | **Tertile 1** | **Tertile 2** | **Tertile 3** |
| N | 4184 | 1395 | 1394 | 1395 | 1395 | 1394 | 1395 | 1395 | 1394 | 1395 |
| Dietary pattern score | 0.0 (1.0) | -0.9 (0.3) | -0.2 (0.2) | 1.1 (0.9) | -0.8 (0.3) | -0.2 (0.2) | 1.0 (1.0) | -0.7 (0.3) | -0.2 (0.1) | 0.9 (1.2) |
| Other fruits | 181.0 (155.8) | 94.0 (65.7) | 162.3 (98.6) | 286.7 (199.3) | 177.2 (166.3) | 169.6 (149.1) | 196.2 (150.3) | 210.1 (191.9) | 160.1 (128.1) | 172.8 (135.3) |
| Potato | 16.6 (23.2) | 7.8 (8.9) | 13.2 (12.3) | 28.7 (34.0) | 15.5 (23.5) | 15.6 (24.4) | 18.6 (21.7) | 13.7 (19.6) | 16.0 (21.7) | 20.0 (27.3) |
| Berries | 60.1 (88.1) | 27.2 (30.4) | 48.5 (46.4) | 104.5 (130.5) | 53.2 (92.6) | 56.6 (78.8) | 70.4 (91.3) | 73.5 (118.3) | 49.4 (58.6) | 57.2 (74.7) |
| Refined grains | 109.2 (90.1) | 67.0 (42.8) | 100.0 (61.3) | 160.6 (119.3) | 114.0 (107.5) | 100.6 (75.9) | 113.0 (83.2) | 86.1 (62.0) | 104.8 (74.4) | 136.8 (116.8) |
| Other vegetables | 124.3 (123.7) | 68.3 (53.6) | 112.6 (84.5) | 191.9 (167.5) | 85.4 (76.9) | 114.0 (95.5) | 173.4 (163.9) | 125.4 (123.8) | 111.5 (107.8) | 135.8 (136.7) |
| Whole grain | 15.8 (31.5) | 5.5 (9.4) | 12.4 (19.3) | 29.6 (47.0) | 15.0 (38.9) | 13.7 (23.1) | 18.7 (30.3) | 15.5 (30.5) | 15.1 (32.7) | 16.9 (31.3) |
| Oranges | 42.6 (70.4) | 18.2 (22.6) | 35.3 (43.3) | 74.4 (104.0) | 52.6 (96.7) | 37.9 (50.6) | 37.4 (52.9) | 51.7 (88.7) | 36.8 (59.7) | 39.3 (57.4) |
| Legumes and legume products | 15.0 (19.4) | 9.0 (9.2) | 13.9 (13.2) | 22.0 (28.0) | 11.0 (11.6) | 13.3 (13.4) | 20.7 (27.7) | 12.6 (13.6) | 14.0 (15.0) | 18.3 (26.5) |
| Nuts | 19.7 (25.5) | 10.2 (13.4) | 19.5 (22.1) | 29.6 (33.1) | 14.8 (19.6) | 18.6 (22.8) | 25.8 (31.3) | 23.5 (30.9) | 16.7 (20.9) | 19.1 (23.1) |
| Leafy vegetables | 80.3 (88.3) | 49.7 (51.5) | 76.8 (69.8) | 114.2 (117.5) | 48.1 (49.8) | 76.4 (68.5) | 116.3 (117.9) | 94.2 (112.6) | 70.6 (71.8) | 76.0 (72.6) |
| Freshwater seafood | 48.2 (56.6) | 44.3 (63.9) | 46.1 (48.7) | 54.2 (55.7) | 20.3 (18.0) | 39.4 (28.7) | 84.9 (79.1) | 64.8 (74.6) | 37.1 (39.5) | 42.7 (45.4) |
| Seafood | 23.8 (36.3) | 24.5 (40.9) | 21.6 (30.3) | 25.4 (37.0) | 7.4 (10.7) | 17.2 (16.5) | 47.0 (52.2) | 27.3 (43.2) | 18.6 (28.9) | 25.6 (35.0) |
| Red meat | 79.7 (78.0) | 69.3 (83.4) | 77.1 (67.2) | 92.7 (80.8) | 40.9 (31.6) | 67.3 (40.9) | 130.9 (106.4) | 68.1 (58.2) | 72.0 (65.0) | 99.0 (100.5) |
| Poultry | 20.8 (30.1) | 18.4 (33.8) | 19.1 (22.1) | 25.0 (32.4) | 8.6 (10.3) | 16.7 (16.3) | 37.2 (43.6) | 16.3 (20.6) | 18.6 (24.6) | 27.5 (40.2) |
| Fungi and algae | 27.6 (37.0) | 18.6 (21.9) | 26.1 (35.6) | 38.2 (46.5) | 16.7 (17.8) | 23.9 (25.1) | 42.1 (53.1) | 24.5 (32.6) | 26.9 (39.9) | 31.4 (37.8) |
| Dairy | 190.7 (156.1) | 161.9 (123.8) | 197.2 (166.1) | 213.1 (169.9) | 139.2 (99.4) | 190.0 (129.2) | 243.0 (202.9) | 246.2 (190.6) | 163.5 (112.6) | 162.5 (139.8) |
| Eggs | 49.3 (34.6) | 42.2 (27.1) | 50.3 (33.8) | 55.5 (40.3) | 38.4 (23.4) | 49.3 (28.7) | 60.4 (44.4) | 60.5 (42.8) | 43.1 (25.9) | 44.4 (29.9) |
| Rice | 141.0 (94.0) | 144.7 (96.4) | 141.2 (94.3) | 137.2 (91.2) | 122.1 (85.7) | 143.0 (93.8) | 157.9 (98.7) | 140.8 (90.6) | 136.7 (89.7) | 145.5 (101.2) |
| Processed meat | 3.8 (9.5) | 2.8 (6.9) | 3.4 (8.2) | 5.1 (12.4) | 3.0 (8.2) | 3.3 (8.4) | 4.9 (11.4) | 0.5 (1.7) | 1.5 (3.0) | 9.3 (14.6) |
| Sauce | 0.9 (3.5) | 0.8 (3.9) | 0.8 (2.7) | 1.2 (3.6) | 1.1 (4.5) | 0.8 (2.8) | 0.9 (2.8) | 0.1 (0.4) | 0.3 (0.8) | 2.4 (5.6) |
| Tea | 2.3 (6.2) | 1.6 (3.8) | 2.5 (6.5) | 2.9 (7.7) | 2.7 (8.2) | 2.1 (4.9) | 2.2 (5.0) | 0.4 (1.2) | 1.2 (2.4) | 5.5 (9.7) |
| Coffee | 11.5 (58.0) | 18.8 (77.4) | 8.1 (49.0) | 7.7 (40.4) | 6.4 (35.2) | 8.5 (35.1) | 19.7 (86.8) | 0.6 (4.0) | 2.1 (10.1) | 31.9 (96.7) |
| Pickled vegetables | 2.9 (21.3) | 5.8 (33.1) | 1.8 (13.4) | 1.0 (8.5) | 0.5 (5.2) | 2.7 (15.6) | 5.5 (32.9) | 0.1 (1.0) | 0.4 (3.0) | 8.2 (36.2) |
| Fried food | 2.1 (7.6) | 1.4 (4.2) | 2.0 (5.8) | 2.8 (11.0) | 2.3 (9.4) | 1.8 (5.9) | 2.1 (7.0) | 0.4 (1.4) | 1.0 (2.6) | 4.8 (12.3) |
| Pastry | 6.2 (17.8) | 2.8 (5.9) | 5.2 (9.9) | 10.5 (28.1) | 7.1 (25.5) | 5.2 (10.9) | 6.3 (13.6) | 2.3 (6.2) | 4.5 (8.6) | 11.7 (28.1) |

Data are presented as mean (standard deviation, SD) in grams/day.

### Table S6. Differences in food intake per one unit of dietary pattern score

|  | **Plant-based**  **dietary pattern** | **Animal-based**  **dietary pattern** | **Processed food and beverage**  **dietary pattern** |
| --- | --- | --- | --- |
| Other fruits | 95.23 (91.49, 98.97) | 7.87 (3.15, 12.58) | -5.81 (-10.53, -1.09) |
| Potato | 11.96 (11.35, 12.56) | 1.75 (1.05, 2.45) | 2.80 (2.10, 3.50) |
| Berries | 43.01 (40.68, 45.34) | 6.56 (3.89, 9.22) | -4.89 (-7.55, -2.22) |
| Refined grains | 43.23 (40.84, 45.63) | 0.63 (-2.10, 3.36) | 20.02 (17.36, 22.68) |
| Other vegetables | 55.25 (51.89, 58.60) | 40.91 (37.37, 44.45) | 10.13 (6.39, 13.86) |
| Whole grain | 13.61 (12.75, 14.47) | 1.24 (0.28, 2.19) | 1.11 (0.15, 2.06) |
| Oranges | 30.25 (28.33, 32.18) | -6.84 (-8.97, -4.72) | -3.07 (-5.20, -0.94) |
| Legumes and legume products | 6.46 (5.90, 7.01) | 4.52 (3.95, 5.10) | 2.72 (2.14, 3.30) |
| Nuts | 8.18 (7.45, 8.91) | 4.56 (3.80, 5.32) | -1.00 (-1.77, -0.23) |
| Leafy vegetables | 28.33 (25.79, 30.86) | 28.52 (25.99, 31.06) | -2.95 (-5.63, -0.28) |
| Freshwater seafood | 3.28 (1.57, 5.00) | 37.59 (36.30, 38.87) | -6.72 (-8.42, -5.01) |
| Seafood | -0.27 (-1.37, 0.84) | 23.50 (22.66, 24.35) | 1.42 (0.32, 2.52) |
| Red meat | 8.49 (6.13, 10.84) | 45.82 (43.91, 47.74) | 15.43 (13.11, 17.75) |
| Poultry | 2.76 (1.86, 3.67) | 16.50 (15.74, 17.26) | 6.42 (5.53, 7.31) |
| Fungi and algae | 8.23 (7.14, 9.33) | 11.41 (10.34, 12.48) | 3.82 (2.70, 4.93) |
| Dairy | 19.39 (14.69, 24.08) | 43.42 (38.88, 47.97) | -25.17 (-29.84, -20.50) |
| Eggs | 5.41 (4.38, 6.45) | 8.30 (7.28, 9.32) | -4.94 (-5.98, -3.90) |
| Rice | -3.39 (-6.24, -0.55) | 13.72 (10.90, 16.54) | 1.59 (-1.26, 4.44) |
| Processed meat | 1.10 (0.81, 1.39) | 0.94 (0.65, 1.22) | 5.27 (5.03, 5.51) |
| Sauce | 0.13 (0.02, 0.23) | -0.05 (-0.16, 0.05) | 1.60 (1.51, 1.69) |
| Tea | -6.25 (-8.00, -4.50) | 6.72 (4.98, 8.47) | 26.29 (24.73, 27.86) |
| Coffee | -2.98 (-3.62, -2.34) | 2.41 (1.77, 3.05) | 9.26 (8.68, 9.84) |
| Pickled vegetables | 0.42 (0.23, 0.61) | -0.32 (-0.51, -0.13) | 2.62 (2.45, 2.79) |
| Fried food | 0.67 (0.44, 0.90) | -0.17 (-0.40, 0.06) | 2.87 (2.66, 3.08) |
| Pastry | 4.28 (3.75, 4.80) | -0.41 (-0.95, 0.13) | 5.19 (4.68, 5.71) |

Data were presented as beta (95% confidence interval, 95%CI) from the univariate linear model.

### Table S7. Associations between three dietary patterns and small for gestational age and large for gestational age among pregnant women in Shanghai, China.

|  | **Score category** | | | |
| --- | --- | --- | --- | --- |
| **Variables** | **Tertile 1** | **Tertile 2** | **Tertile 3** | ***P*-trend** |
| **Small for gestational age** |  |  |  |  |
| Plant-based dietary pattern |  |  |  |  |
| N Cases/Total | 87/1183 | 89/1159 | 71/1139 |  |
| Model 1 | 1.00 (ref) | 1.06 (0.78-1.44) | 0.84 (0.61-1.16) | 0.263 |
| Model 2 | 1.00 (ref) | 1.02 (0.74-1.40) | 0.76 (0.51-1.12) | 0.143 |
| Model 3 | 1.00 (ref) | 1.02 (0.74-1.40) | 0.76 (0.51-1.13) | 0.172 |
| Animal-based dietary pattern |  |  |  |  |
| N Cases/Total | 86/1172 | 79/1136 | 82/1173 |  |
| Model 1 | 1.00 (ref) | 0.98 (0.71-1.34) | 0.98 (0.71-1.34) | 0.901 |
| Model 2 | 1.00 (ref) | 0.99 (0.71-1.37) | 0.99 (0.69-1.41) | 0.949 |
| Model 3 | 1.00 (ref) | 0.96 (0.69-1.34) | 0.94 (0.64-1.36) | 0.865 |
| Processed food and beverage  dietary pattern |  |  |  |  |
| N Cases/Total | 85/1173 | 75/1165 | 87/1143 |  |
| Model 1 | 1.00 (ref) | 0.89 (0.65-1.23) | 1.07 (0.78-1.46) | 0.545 |
| Model 2 | 1.00 (ref) | 0.87 (0.63-1.21) | 1.15 (0.84-1.59) | 0.274 |
| Model 3 | 1.00 (ref) | 0.86 (0.62-1.20) | 1.12 (0.80-1.55) | 0.351 |
| **Large for gestational age** |  |  |  |  |
| Plant-based dietary pattern |  |  |  |  |
| N Cases/Total | 211/1307 | 234/1304 | 256/1324 |  |
| Model 1 | 1.00 (ref) | 1.13 (0.92-1.39) | 1.25 (1.02-1.52) | 0.034 |
| Model 2 | 1.00 (ref) | 1.11 (0.90-1.37) | 1.13 (0.89-1.43) | 0.354 |
| Model 3 | 1.00 (ref) | 1.10 (0.89-1.36) | 1.12 (0.88-1.43) | 0.353 |
| Animal-based dietary pattern |  |  |  |  |
| N Cases/Total | 223/1309 | 257/1314 | 221/1312 |  |
| Model 1 | 1.00 (ref) | 1.16 (0.95-1.42) | 0.97 (0.79-1.18) | 0.542 |
| Model 2 | 1.00 (ref) | 1.16 (0.94-1.42) | 0.91 (0.72-1.14) | 0.293 |
| Model 3 | 1.00 (ref) | 1.17 (0.95-1.44) | 0.93 (0.73-1.18) | 0.393 |
| Processed food and beverage  dietary pattern |  |  |  |  |
| N Cases/Total | 222/1310 | 229/1319 | 250/1306 |  |
| Model 1 | 1.00 (ref) | 1.02 (0.84-1.26) | 1.16 (0.95-1.42) | 0.124 |
| Model 2 | 1.00 (ref) | 1.03 (0.84-1.26) | 1.05 (0.85-1.29) | 0.672 |
| Model 3 | 1.00 (ref) | 1.03 (0.84-1.27) | 1.06 (0.85-1.30) | 0.714 |

ORs and 95% CIs were calculated in logistic model: model 1 adjusted for maternal age (continuous variables, in years), and infant sex (male or female); model 2 also included maternal domicile place (south China, north China, or Shanghai surrounding area), pre-pregnancy BMI (<18.5, 18.5-23.9, or ≥24.0 kg/m^2^), household income (<100,000, 100,000-350,000, or ≥350,000 yuan/year), education (<13 or ≥13 years), baseline season (spring/winter, summer, or autumn), parity (primiparous or multiparous), gestational periods at recruitment (first, second, or third trimester), passive smoking (yes or no), alcohol drinking (yes or no), physical activity (active or inactive), multivitamin (yes or no), calcium tablets(yes or no), folic acid (yes or no), and total energy (continuous variables, in kcal/day) on model 2. Model 3 mutually adjusted for all extracted dietary scores in the same model based on model 2.

*P* for trend was obtained by modelling the median value of the tertiles into the logistic regression models.

N Cases/Total=number of cases and total participants in the study. OR=odds ratio. CI=confidence interval. Ref= reference.

### Table S8. Associations between selected food groups with high loading (r>0.4) in plant-based dietary pattern and macrosomia

| **Food** | **Tertile 1** | **Tertile 2** | **Tertile 3** | ***P*-trend** |
| --- | --- | --- | --- | --- |
| **Other fruits** |  |  |  |  |
| N Cases/Total | 75/1362 | 71/1357 | 73/1360 |  |
| Adjusted OR | 1.00 | 0.88 (0.62-1.25) | 0.84 (0.59-1.20) | 0.371 |
| **Potato** |  |  |  |  |
| N Cases/Total | 59/1410 | 70/1437 | 90/1232 |  |
| Adjusted OR | 1.00 | 1.18 (0.82-1.70) | 1.72 (1.20-2.47) | 0.002 |
| **Berries** |  |  |  |  |
| N Cases/Total | 64/1359 | 76/1376 | 79/1344 |  |
| Adjusted OR | 1.00 | 1.24 (0.87-1.77) | 1.29 (0.89-1.86) | 0.246 |
| **Refined grain** |  |  |  |  |
| N Cases/Total | 60/1356 | 76/1365 | 83/1358 |  |
| Adjusted OR | 1.00 | 1.23 (0.86-1.76) | 1.22 (0.83-1.80) | 0.380 |
| **Other vegetables** |  |  |  |  |
| N Cases/Total | 70/1362 | 66/1351 | 83/1366 |  |
| Adjusted OR | 1.00 | 0.96 (0.67-1.37) | 1.15 (0.79-1.65) | 0.382 |
| **Whole grains** |  |  |  |  |
| N Cases/Total | 74/1352 | 78/1657 | 67/1070 |  |
| Adjusted OR | 1.00 | 0.89 (0.63-1.25) | 1.18 (0.81-1.70) | 0.229 |
| **Oranges** |  |  |  |  |
| N Cases/Total | 73/1419 | 58/1305 | 88/1355 |  |
| Adjusted OR | 1.00 | 0.86 (0.60-1.24) | 1.16 (0.82-1.64) | 0.231 |

Adjusted ORs and 95% CIs were calculated in logistic model adjusted for maternal age (continuous variables, in years), and infant sex (male, female), maternal domicile place (south China, north China, or Shanghai surrounding area), pre-pregnancy BMI (<18.5, 18.5-23.9, or ≥24 kg/m^2^), household income (<100,000, 100,000-350,000, or ≥350,000 yuan/year), education (<13 or ≥13 years), baseline season (spring/winter, summer, or autumn), parity (primiparous or multiparous), gestational periods at recruitment (first, second, or third trimester), passive smoking (yes or no), alcohol drinking (yes or no), physical activity (active or inactive), multivitamin (yes or no), calcium tablets(yes or no), folic acid (yes or no), total energy (continuous variables, in kcal/day), gestational week at birth (continuous variables, in weeks), and the other two dietary scores.

*P* for trend was obtained by modelling the median value of the tertiles into the logistic regression models.

N Cases/Total=number of cases and total participants in the study. OR=odds ratio. CI=confidence interval. Ref= reference.

### Table S9. Associations of dietary patterns with birth weight traits among spontaneous labors (N=2,231)

|  | **Score category** | | | |
| --- | --- | --- | --- | --- |
| **Variables** | **Tertile 1** | **Tertile 2** | **Tertile 3** | ***P*-trend** |
| **Low birth weight** |  |  |  |  |
| Plant-based dietary pattern |  |  |  |  |
| N Cases/Total | 15/715 | 20/720 | 16/713 |  |
| Adjusted OR | 1.00 (ref) | 0.98 (0.42-2.28) | 0.40 (0.14-1.17) | 0.083 |
| Animal-based dietary pattern |  |  |  |  |
| N Cases/Total | 19/730 | 13/701 | 19/717 |  |
| Adjusted OR | 1.00 (ref) | 0.79 (0.31-1.97) | 1.10 (0.39-3.06) | 0.892 |
| Processed food and beverage dietary pattern |  |  |  |  |
| N Cases/Total | 19/718 | 17/701 | 15/729 |  |
| Adjusted OR | 1.00 (ref) | 1.16 (0.50-2.66) | 0.46 (0.17-1.24) | 0.097 |
| **Macrosomia** |  |  |  |  |
| Plant-based dietary pattern |  |  |  |  |
| N Cases/Total | 21/721 | 29/729 | 33/730 |  |
| Adjusted OR | 1.00 (ref) | 1.36 (0.75-2.48) | 1.40 (0.73-2.69) | 0.393 |
| Animal-based dietary pattern |  |  |  |  |
| N Cases/Total | 25/736 | 34/722 | 24/722 |  |
| Adjusted OR | 1.00 (ref) | 1.64 (0.92-2.90) | 1.07 (0.54-2.09) | 0.944 |
| Processed food and beverage dietary pattern |  |  |  |  |
| N Cases/Total | 25/724 | 21/705 | 37/751 |  |
| Adjusted OR | 1.00 (ref) | 0.76 (0.41-1.41) | 1.23 (0.70-2.15) | 0.315 |

Adjusted ORs and 95% CIs were calculated in logistic model adjusted for maternal age (continuous variables, in years), and infant sex (male or female), maternal domicile place (south China, north China, or Shanghai surrounding area), pre-pregnancy BMI (<18.5, 18.5-23.9, or ≥24.0 kg/m^2^), household income (<100,000, 100,000-350,000, or ≥350,000 yuan/year), education (<13 or ≥13 years), baseline season (spring/winter, summer, or autumn), parity (primiparous or multiparous), gestational periods at recruitment (first, second, or third trimester), passive smoking (yes or no), alcohol drinking (yes or no), physical activity (active or inactive), multivitamin (yes or no), calcium tablets(yes or no), folic acid (yes or no), total energy (continuous variables, in kcal/day), gestational week at birth (continuous variables, in weeks), and the other two dietary scores in the same model based on model 2.

*P* for trend was obtained by modelling the median value of the tertiles into the logistic regression models.

N Cases/Total=number of cases and total participants in the study. OR=odds ratio. CI=confidence interval. Ref= reference.

### Table S10. Associations of dietary patterns with birth weight traits among sample in term (born between 37 to <42 completed weeks, N=4,001)

|  | **Score category** | | | |
| --- | --- | --- | --- | --- |
| **Variables** | **Tertile 1** | **Tertile 2** | **Tertile 3** | ***P*-trend** |
| **Low birth weight** |  |  |  |  |
| Plant-based dietary pattern |  |  |  |  |
| N Cases/Total | 16/1280 | 11/1251 | 14/1254 |  |
| Adjusted OR | 1.00 (ref) | 0.52 (0.23-1.17) | 0.59 (0.24-1.43) | 0.267 |
| Animal-based dietary pattern |  |  |  |  |
| N Cases/Total | 8/1277 | 16/1240 | 17/1268 |  |
| Adjusted OR | 1.00 (ref) | 2.10 (0.86-5.12) | 2.07 (0.78-5.50) | 0.258 |
| Processed food and beverage dietary pattern |  |  |  |  |
| N Cases/Total | 17/1249 | 15/1277 | 9/1259 |  |
| Adjusted OR | 1.00 (ref) | 0.93 (0.45-1.95) | 0.48 (0.20-1.13) | 0.084 |
| **Macrosomia** |  |  |  |  |
| Plant-based dietary pattern |  |  |  |  |
| N Cases/Total | 53/1317 | 79/1319 | 84/1324 |  |
| Adjusted OR | 1.00 (ref) | 1.47 (1.02-2.14) | 1.52 (1.00-2.31) | 0.079 |
| Animal-based dietary pattern |  |  |  |  |
| N Cases/Total | 59/1328 | 90/1314 | 67/1318 |  |
| Adjusted OR | 1.00 (ref) | 1.72 (1.20-2.46) | 1.22 (0.80-1.84) | 0.507 |
| Processed food and beverage dietary pattern |  |  |  |  |
| N Cases/Total | 75/1307 | 61/1323 | 80/1330 |  |
| Adjusted OR | 1.00 (ref) | 0.84 (0.59-1.20) | 1.08 (0.77-1.53) | 0.639 |

Adjusted ORs and 95% CIs were calculated in logistic model adjusted for maternal age (continuous variables, in years), and infant sex (male or female), maternal domicile place (south China, north China, or Shanghai surrounding area), pre-pregnancy BMI (<18.5, 18.5-23.9, or ≥24.0 kg/m^2^), household income (<100,000, 100,000-350,000, or ≥350,000 yuan/year), education (<13 or ≥13 years), baseline season (spring/winter, summer, or autumn), parity (primiparous or multiparous), gestational periods at recruitment (first, second, or third trimester), passive smoking (yes or no), alcohol drinking (yes or no), physical activity (active or inactive), multivitamin (yes or no), calcium tablets(yes or no), folic acid (yes or no), total energy (continuous variables, in kcal/day), gestational week at birth (continuous variables, in weeks), and the other two dietary scores in the same model based on model 2.

*P* for trend was obtained by modelling the median value of the tertiles into the logistic regression models.

N Cases/Total=number of cases and total participants in the study. OR=odds ratio. CI=confidence interval. Ref= reference.

### Table S11. Associations of dietary patterns with birth weight traits after excluding participants who were less than six weeks pregnant (N=4,153)

|  | **Score category** | | | |
| --- | --- | --- | --- | --- |
| **Variables** | **Tertile 1** | **Tertile 2** | **Tertile 3** | ***P*-trend** |
| **Low birth weight** |  |  |  |  |
| Plant-based dietary pattern |  |  |  |  |
| N Cases/Total | 36/1324 | 32/1307 | 37/1306 |  |
| Adjusted OR | 1.00 (ref) | 0.80 (0.45-1.43) | 0.76 (0.38-1.51) | 0.410 |
| Animal-based dietary pattern |  |  |  |  |
| N Cases/Total | 28/1323 | 38/1291 | 39/1323 |  |
| Adjusted OR | 1.00 (ref) | 1.56 (0.85-2.87) | 1.43 (0.71-2.87) | 0.422 |
| Processed food and beverage dietary pattern |  |  |  |  |
| N Cases/Total | 44/1307 | 32/1325 | 29/1305 |  |
| Adjusted OR | 1.00 (ref) | 0.84 (0.48-1.46) | 0.58 (0.32-1.07) | 0.069 |
| **Macrosomia** |  |  |  |  |
| Plant-based dietary pattern |  |  |  |  |
| N Cases/Total | 53/1341 | 78/1353 | 85/1354 |  |
| Adjusted OR | 1.00 (ref) | 1.46 (1.01-2.12) | 1.58 (1.04-2.40) | 0.051 |
| Animal-based dietary pattern |  |  |  |  |
| N Cases/Total | 60/1355 | 90/1343 | 66/1350 |  |
| Adjusted OR | 1.00 (ref) | 1.70 (1.19-2.43) | 1.20 (0.79-1.82) | 0.536 |
| Processed food and beverage  dietary pattern |  |  |  |  |
| N Cases/Total | 77/1340 | 59/1352 | 80/1356 |  |
| Adjusted OR | 1.00 (ref) | 0.79 (0.55-1.14) | 1.07 (0.76-1.51) | 0.653 |

Adjusted ORs and 95% CIs were calculated in logistic model adjusted for maternal age (continuous variables, in years), and infant sex (male or female), maternal domicile place (south China, north China, or Shanghai surrounding area), pre-pregnancy BMI (<18.5, 18.5-23.9, or ≥24.0 kg/m^2^), household income (<100,000, 100,000-350,000, or ≥350,000 yuan/year), education (<13 or ≥13 years), baseline season (spring/winter, summer, or autumn), parity (primiparous or multiparous), gestational periods at recruitment (first, second, or third trimester), passive smoking (yes or no), alcohol drinking (yes or no), physical activity (active or inactive), multivitamin (yes or no), calcium tablets(yes or no), folic acid (yes or no), total energy (continuous variables, in kcal/day), gestational week at birth (continuous variables, in weeks), and the other two dietary scores in the same model based on model 2.

*P* for trend was obtained by modelling the median value of the tertiles into the logistic regression models.

N Cases/Total=number of cases and total participants in the study. OR=odds ratio. CI=confidence interval. Ref= reference.

### Table S12. Associations of dietary patterns with birth weight traits using multiple imputations with chained equations to assign missing values of covariates (N=4,184)

|  | **Score category** | | | |
| --- | --- | --- | --- | --- |
| **Variables** | **Tertile 1** | **Tertile 2** | **Tertile 3** | ***P*-trend** |
| **Low birth weight** |  |  |  |  |
| Plant-based dietary pattern |  |  |  |  |
| N Cases/Total | 36/1341 | 32/1315 | 37/1309 |  |
| Adjusted OR | 1.00 (ref) | 0.81 (0.45-1.44) | 0.77 (0.39-1.53) | 0.441 |
| Animal-based dietary pattern |  |  |  |  |
| N Cases/Total | 28/1335 | 38/1303 | 39/1327 |  |
| Adjusted OR | 1.00 (ref) | 1.57 (0.85-2.89) | 1.45 (0.72-2.92) | 0.399 |
| Processed food and beverage dietary pattern |  |  |  |  |
| N Cases/Total | 44/1318 | 32/1333 | 29/1314 |  |
| Adjusted OR | 1.00 (ref) | 0.85 (0.49-1.49) | 0.59 (0.32-1.09) | 0.077 |
| **Macrosomia** |  |  |  |  |
| Plant-based dietary pattern |  |  |  |  |
| N Cases/Total | 54/1359 | 79/1362 | 86/1358 |  |
| Adjusted OR | 1.00 (ref) | 1.45 (1.00-2.10) | 1.55 (1.03-2.35) | 0.062 |
| Animal-based dietary pattern |  |  |  |  |
| N Cases/Total | 60/1367 | 91/1356 | 68/1356 |  |
| Adjusted OR | 1.00 (ref) | 1.71 (1.20-2.44) | 1.21 (0.80-1.82) | 0.531 |
| Processed food and beverage  dietary pattern |  |  |  |  |
| N Cases/Total | 77/1351 | 61/1362 | 81/1366 |  |
| Adjusted OR | 1.00 (ref) | 0.83 (0.58-1.18) | 1.08 (0.76-1.52) | 0.652 |

Adjusted ORs and 95% CIs were calculated in logistic model adjusted for maternal age (continuous variables, in years), and infant sex (male or female), maternal domicile place (south China, north China, or Shanghai surrounding area), pre-pregnancy BMI (<18.5, 18.5-23.9, or ≥24.0 kg/m^2^), household income (<100,000, 100,000-350,000, or ≥350,000 yuan/year), education (<13 or ≥13 years), baseline season (spring/winter, summer, or autumn), parity (primiparous or multiparous), gestational periods at recruitment (first, second, or third trimester), passive smoking (yes or no), alcohol drinking (yes or no), physical activity (active or inactive), multivitamin (yes or no), calcium tablets(yes or no), folic acid (yes or no), total energy (continuous variables, in kcal/day), gestational week at birth (continuous variables, in weeks), and the other two dietary scores in the same model based on model 2.

*P* for trend was obtained by modelling the median value of the tertiles into the logistic regression models.

N Cases/Total=number of cases and total participants in the study. OR=odds ratio. CI=confidence interval. Ref= reference.


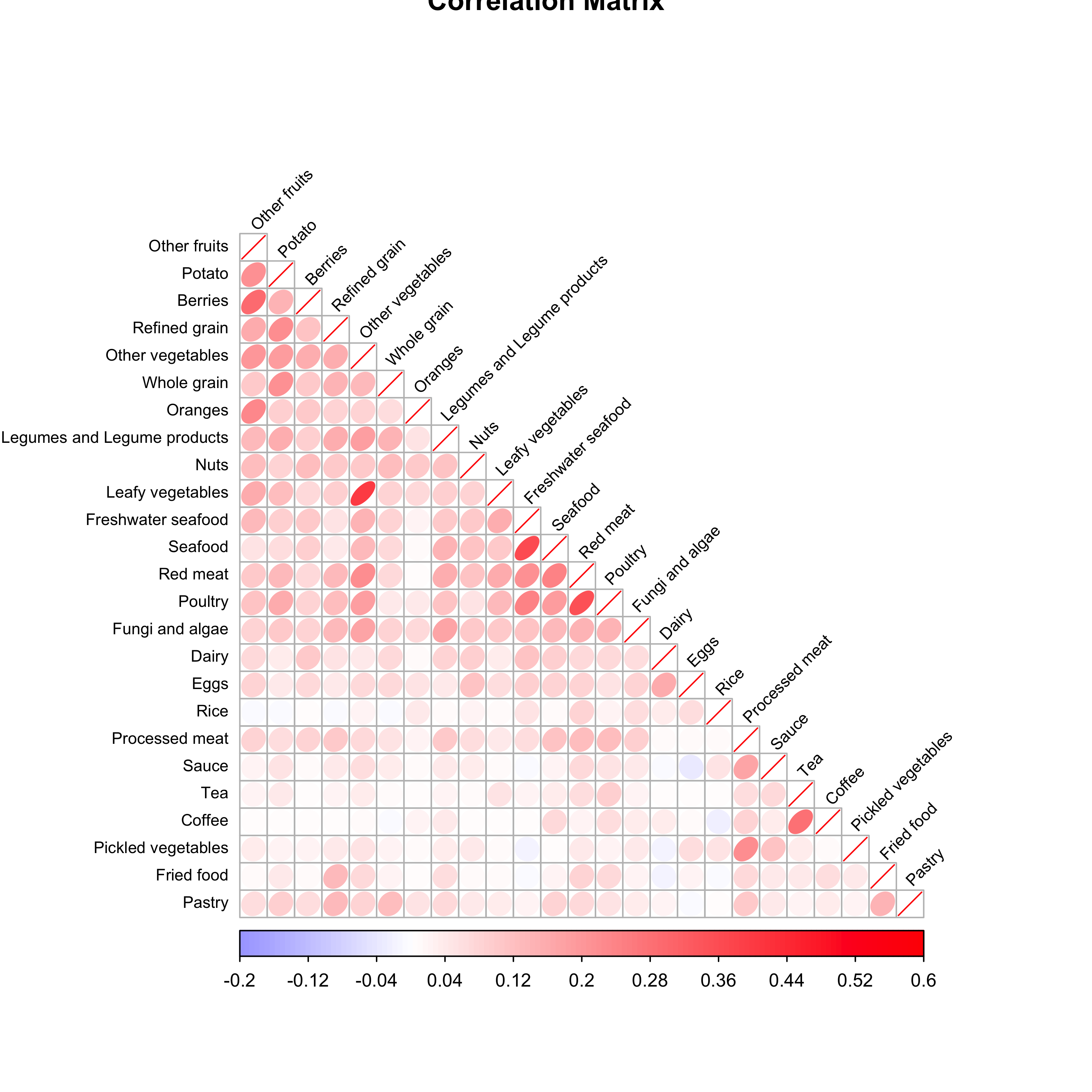


### Figure S1. Correlations for the 25 food groups used for generating dietary patterns.
